# Genome-wide binding of SEPALLATA3 and AGAMOUS complexes determined by sequential DNA-affinity purification sequencing

**DOI:** 10.1093/nar/gkaa729

**Published:** 2020-09-05

**Authors:** Xuelei Lai, Arnaud Stigliani, Jérémy Lucas, Véronique Hugouvieux, François Parcy, Chloe Zubieta

**Affiliations:** Laboratoire de Physiologie Cellulaire et Végétale, Université Grenoble-Alpes, CNRS, CEA, INRAE, IRIG-DBSCI, 38000 Grenoble, France; Laboratoire de Physiologie Cellulaire et Végétale, Université Grenoble-Alpes, CNRS, CEA, INRAE, IRIG-DBSCI, 38000 Grenoble, France; Biotech Research and Innovation Centre, University of Copenhagen, Copenhagen, DK-2200, Denmark; Department of Biology, University of Copenhagen, Copenhagen, DK-2200 Denmark; Laboratoire de Physiologie Cellulaire et Végétale, Université Grenoble-Alpes, CNRS, CEA, INRAE, IRIG-DBSCI, 38000 Grenoble, France; Laboratoire de Physiologie Cellulaire et Végétale, Université Grenoble-Alpes, CNRS, CEA, INRAE, IRIG-DBSCI, 38000 Grenoble, France; Laboratoire de Physiologie Cellulaire et Végétale, Université Grenoble-Alpes, CNRS, CEA, INRAE, IRIG-DBSCI, 38000 Grenoble, France; Laboratoire de Physiologie Cellulaire et Végétale, Université Grenoble-Alpes, CNRS, CEA, INRAE, IRIG-DBSCI, 38000 Grenoble, France

## Abstract

The MADS transcription factors (TF), SEPALLATA3 (SEP3) and AGAMOUS (AG) are required for floral organ identity and floral meristem determinacy. While dimerization is obligatory for DNA binding, SEP3 and SEP3–AG also form tetrameric complexes. How homo and hetero-dimerization and tetramerization of MADS TFs affect genome-wide DNA-binding and gene regulation is not known. Using sequential DNA affinity purification sequencing (seq-DAP-seq), we determined genome-wide binding of SEP3 homomeric and SEP3–AG heteromeric complexes, including SEP3^Δtet^-AG, a complex with a SEP3 splice variant, SEP3^Δtet^, which is largely dimeric and SEP3–AG tetramer. SEP3 and SEP3–AG share numerous bound regions, however each complex bound unique sites, demonstrating that protein identity plays a role in DNA-binding. SEP3–AG and SEP3^Δtet^-AG share a similar genome-wide binding pattern; however the tetrameric form could access new sites and demonstrated a global increase in DNA-binding affinity. Tetramerization exhibited significant cooperative binding with preferential distances between two sites, allowing efficient binding to regions that are poorly recognized by dimeric SEP3^Δtet^-AG. By intersecting seq-DAP-seq with ChIP-seq and expression data, we identified unique target genes bound either in SEP3–AG seq-DAP-seq or in SEP3/AG ChIP-seq. Seq-DAP-seq is a versatile genome-wide technique and complements *in vivo* methods to identify putative direct regulatory targets.

## INTRODUCTION

Transcription factors (TF) often act cooperatively by forming heteromeric complexes, with proper oligomerization patterns thought to be a key component of gene regulation (1). How oligomerization affects DNA-binding site selection, either via changes in affinity or specificity, has been difficult to determine. The MADS TFs, named for founding members MCM1 (*Saccharomyces cerevisiae*), AGAMOUS (*Arabidopsis thaliana*), DEFICIENS (*Antirrhinum majus*) and Serum response factor (*Homo sapiens*), provide a key example of a TF family that is present in almost all eukaryotes and binds a highly conserved DNA sequence called a CArG-box (CC-’Adenine-rich’-GG) as an obligate dimer (2–4). In seed plants, however, this family has undergone a significant expansion and is able to form ternary complexes via the incorporation of protein–protein interaction domains present in the ‘MIKC’ MADS TFs, with MIKC referring to the domain names (5–7). The canonical M, or MADS DNA-binding domain (DBD) of the MADS TFs, is diversified via the I (Intervening), K (Keratin-like) and C-terminal domains, with the K domain acting as a plant-specific module required for tetramer formation within the MADS family. It is hypothesized that the K domain, was critical in the functional diversification of the family in plants by allowing the binding or accessing of novel genomic sites (8). However, this hypothesis has been challenging to test.

Some of the most extensively studied MADS TF complexes are those involved in flower organ identity. Their roles are described in the ‘floral quartet model,’ which explains the function of different tetrameric MADS complexes in floral organ development (9). This model elegantly synthesizes gene expression and protein–protein interaction patterns to illustrate how different MADS tetramers are required to trigger the regulation of unique subsets of target genes which in turn determine the identity of the organs in each whorl of the flower. The floral quartet model has been established in multiple species and is a general model for angiosperm floral organ identity dependent on MADS TF tetramerization (for review see (8)). For example, in the model plant *A. thaliana*, the MADS TFs, APETALA1 (AP1), APETALA3 (AP3), PISTILLATA (PI), AGAMOUS (AG) and the SEPALLATA clade (SEP1, 2, 3 and 4) act combinatorially to specify organ identity. The first whorl sepal identity is determined by the tetrameric AP1-SEP complex, petal identity is specified by AP1-SEP-AP3-PI, stamen identity by AP3-PI-SEP-AG and carpel identity by tetrameric SEP-AG. These unique MADS complexes exhibit slightly altered DNA binding specificity, resulting in the expression and/or repression of different sets of target genes (10–14). In addition to presenting two distinct DBDs and selecting for unique single sites, tetramerization is believed to affect DNA-binding via the formation of DNA loops, as the tetrameric MADS complexes bind two genomic sites independently, looping the DNA between sites (15–17). This adds the possibility of intersite spacing as a criterion for MADS TF complex binding in addition to simple motif selection based on nucleotide sequence of a single site. Thus, determining where in the genome specific MADS TF dimers and tetramers bind is complicated by the promiscuity of MADS protein–protein complex formation within the TF family, the oligomerization state of the proteins and the similar DNA-motifs recognized. For these reasons, deciphering the gene regulatory networks (GRN) dependent on MADS TFs has been a major challenge.

Based on structural and mutagenesis studies, the tetramerization domain of MADS TFs acts independently of the DBD and tetramerization can be abrogated without affecting the ability of the proteins to bind DNA as dimers or to recognize their cognate binding motifs (18,19). Recently, *in planta* experiments targeting the tetramerization interface of SEP3, however, have demonstrated that tetramerization plays an important functional role in the fourth whorl organ formation and floral meristem determinacy (20). *In vitro* electrophoretic mobility shift assay (EMSA) experiments also suggested that tetrameric complexes are able to access certain sites, which dimeric complexes do not bind, although these experiments are based on only a few sequences (20). The relative contributions of homo- and hetero-dimerization and tetramerization to DNA-binding affinity and specificity genome-wide are unknown. The MADS TF complexes provide an ideal model to investigate DNA-binding specificity and affinity and to decipher the role of oligomerization in DNA-binding in the context of the regulation of flower development genes. A new high-throughput sequencing technique using genomic DNA and *in vitro* purified TFs, DNA-affinity purification sequencing (DAP-seq) (21,22), provides the ideal method to address and answer these questions.

We developed a modified DAP-seq method for MADS TFs we term sequential DAP-seq (seq-DAP-seq). Using sequential purification based on multiple tags, we specifically isolate heteromeric MADS TF complexes prior to DNA-affinity purification. This facilitates the identification of binding sites corresponding to a single TF complex as opposed to *in vivo* methods, which will sample all possible physiological complexes containing the target TF. Using this method, the genome-wide binding of the SEP3 homomeric complex, the SEP3–AG heterotetramer and the SEP3^Δtet^-AG heterodimer were determined. These data show that SEP3 and SEP3–AG share many binding sites, but that SEP3–AG is able to access additional genomic regions. Comparing SEP3–AG and SEP3^Δtet^-AG demonstrates that tetramerization results in a preference for specific CArG-box intersite spacing as well as globally increasing in DNA-binding affinity. Comparing seq-DAP-seq with ChIP-seq and expression data for AG and SEP3 allowed the identification of putative *in vivo* direct targets of the SEP3–AG complex. Thus, seq-DAP-seq acts as both an alternative and as a complementary method to ChIP-seq for identifying genome-wide binding sites and putative target genes for the SEP3–AG MADS TF complexes. More broadly, these studies demonstrate the efficacy and simplicity of seq-DAP-seq in determining genome-wide binding of specific heteromeric complexes and identifying a large percentage of potential regulated *in vivo* targets.

## MATERIALS AND METHODS

### Plasmid construction

SEP3 (At1g24260.2), SEP3^Δtet^ (At1g24260.3), a SEP3 splice variant with deletion of amino acids 161–174 and AG (AT4g18960.1) coding sequences were polymerase chain reaction (PCR) amplified and cloned into modified pTnT vectors (Promega) to generate the following C-terminal-tagged constructs: SEP3–3×FLAG, SEP3^Δtet^ -3×FLAG and AG-5×MyC.

### Seq-DAP-seq

*In vitro* protein production was carried out using TnT^®^ SP6 High-Yield Wheat Germ Protein Expression System (Promega L3260) according to the manufacturer's instructions. For single protein DAP-seq, i.e. SEP3, 2 μg input plasmid DNA was used in a 50 μl TnT reaction. For seq-DAP-seq, equal molar amounts of purified plasmid DNA (around 2 μg per construct) were used as input in a 50 μl TnT reaction. For protein complex purification, the 50 μl TnT reaction producing SEP3–AG or SEP3^Δtet^-AG was combined with 50 μl IP buffer (phosphate-buffered saline supplemented with 0.005% NP40 and proteinase inhibitors (Roche)) and mixed with 20 μl anti-FLAG magnetic beads (Merck Millipore). Following 1 h incubation at room temperature, the anti-FLAG magnetic beads were immobilized, and washed three times with 100 μl IP buffer. Protein complexes were eluted with 100 μl IP buffer supplemented with 200 μg/ml 3×FLAG peptide (Merck Millipore). The eluted protein was then immobilized on anti-c-Myc magnetic beads (Thermo Scientific) and washed three times with 100 μl IP buffer in order to isolate homogeneous SEP3–AG or SEP3^Δtet^-AG complexes. In order to verify successful TnT protein production, Western blots of the samples after each immunoprecipitation step were run. The purified protein complex (Supplementary Figure S1), while still bound on anti-c-Myc magnetic beads, was used for DAP-seq following published protocols with minor modifications (22). Briefly, anti-c-Myc beads with bound SEP3–AG or SEP3^Δtet^-AG were resuspended in 100 μl IP buffer, and 50 ng DAP-seq input library pre-ligated with Illumina adaptor sequences was added (the average size of the sheared Col-0 Arabidopsis genomic DNA was 200–400 bp). The reaction was incubated for 90 mins and then washed six times using 100 μl IP buffer. The bound DNA was heated to 98°C for 10 min and eluted in 30 μl EB buffer (10 mM Tris–Cl, pH 8.5). The eluted DNA fragments were PCR amplified using Illumina TruSeq primers for 20 cycles, and purified by AMPure XP beads (Beckman). Due to the higher elution efficiency in our hands using FLAG versus Myc peptides, the anti-FLAG immunoprecipitation step was performed first for all heteromeric complexes. The libraries were quantified by qPCR using NEBNext Library Quant Kit for Illumina following manufacturer's instructions. Libraries with different barcodes were pooled with equal molarity, and sequenced on Illumina HiSeq (Genewiz) with specification of paired-end sequencing of 150 cycles. Each library obtained between 10 and 20 million reads. All DAP-seq and seq-DAP-seq experiments were performed in two to four replicates (Supplementary Figure S2).

### Electrophoretic mobility shift assay (EMSA)

EMSA were performed as described (20). The 96-bp DNA probe from the *KNU* promoter containing two CArG box binding sites with spacing shown as underlined was labeled with Cy5 (Eurofins). The first CArG box was predicted to be strongly bound with a score of 0.87 based on transcription factor flexible model (TFFM), with 1.0 being the highest score. Two additional CArG boxes were identified at 45–46 bp from the first site with low scores (0.006 and 0.017). The CArG box spacing was modified with 5 bp deletions or additions between CArG boxes. For EMSAs in Figure 4F, probe extremities were modified to maintain probe size at 96 bp and sequences are given below with CArG boxes underlined. Proteins were produced as described for seq-DAP-seq without further purification.

*KNU* WT sequence: GGTAAGAGAAACATAGAAACCTTCCATGTTTGGCAATTTCATCTTGGAACTTGATTCACTCTCTCTTGTCTTCTTTGTGCATCACAAGAACAACAA

KNU -5: AGAGGGTAAGAGAAACATAGAAACCTTCCATGTTTGGCAATTTCATCTTGGAACTTACTCTCTCTTGTCTTCTTTGTGCATCACAAGAACAACAAA

KNU -10: GAAAGAGGGTAAGAGAAACATAGAAACCTTCCATGTTTGGCAATTTCATCTTGGACTCTCTCTTGTCTTCTTTGTGCATCACAAGAACAACAAATA

KNU +5: AGAGAAACATAGAAACCTTCCATGTTTGGCAATTTCATCTTGGAAGTCAGCTTGATTCACTCTCTCTTGTCTTCTTTGTGCATCACAAGAACAACA

KNU +10: AAACATAGAAACCTTCCATGTTTGGCAATTTCATCTTGGAAGTCAGTAGCTCTTGATTCACTCTCTCTTGTCTTCTTTGTGCATCACAAGAACAAC

For EMSAs in Supplementary Figure S7A, the WT probe was *KNU* WT sequence (96 bp), and the modified probes were constructed without deleting the extremity bases, thus the size ranged from 86 bp (−10 probe), 91 bp (−5 probe), 101 bp (+5 probe) to 106 bp (+10 probe) (Supplementary Figure S7C).

### Bioinformatic analyses

#### DAP-seq and seq-DAP-seq read processing

Read quality was analysed with FastQC (http://www.bioinformatics.babraham.ac.uk/projects/fastqc/). The first nucleotide of each read was trimmed with Trimmomatic (23) with the HEADCROP:1 option, due to its poor quality. 3′ adapters were detected using FastQC and removed with the NGmerge software (24), used with the –a and –q 33 options. The reads were then aligned with bowtie2 (25) on the TAIR10 version of the *A. thaliana* genome (www.arabidopsis.org), devoid of the mitochondrial and the chloroplast genomes, using –X 2000 and –dovetail option. Only the reads reported at a single position (grep –v ‘XS:i:’) with a maximum of two mismatches with the reference genome (grep -e ‘∧@’ -e ‘XM:i:[012][∧0–9]’) were retained. The duplicate reads were removed using the samtools rmdup program.

#### Peak calling

For each sample, peaks were called using MACS2 (https://github.com/jsh58/MACS) with –f BAMPE, -g 120000000 –q 0.0001 –call-summits and the input DNA as control.

#### Consensus peak determination and pooling

For each DAP experiment (SEP3, SEP3–AG or SEP3^Δtet^-AG), consensus peaks between replicates were defined using the Multiple Sample Peak Calling (MSPC) package (cutoff = 10^−4^) (26). MSPC uses the *P*-value from MACS2 to assess the peak reproducibility. Each consensus peak created was scanned for possible subpeaks (identified based on the presence of multiple peak maxima detected by MACS2), split into several peaks if needed and the peak widths were then normalized to ± 200 bp around the peak maximum. For all the resulting peaks, a mean normalized coverage was computed as the mean of the normalized read coverage for each replicate.

#### Comparison between SEP3, SEP3–AG and SEP3^Δtet^-AG

**Figure 1.**
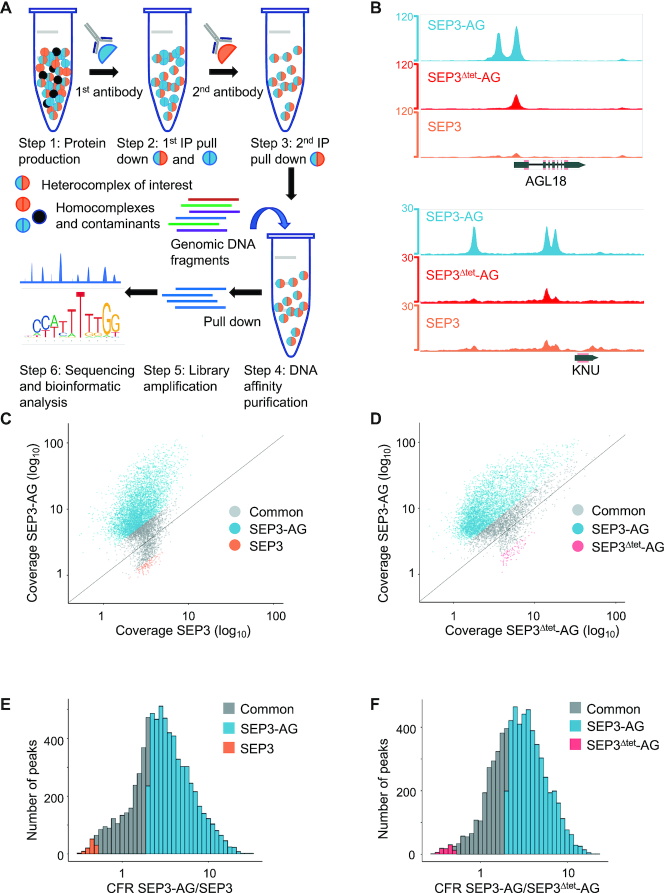
Seq-DAP-seq workflow and data analysis. (**A**) Schematic of the seq-DAP-seq protocol. Each TF or TF combination is expressed with a unique tag and purified by sequential pull-down using an appropriate antibody. Purified TF complexes are used for DNA-affinity purification and sequencing according to published protocols (21). (**B**) Integrated Genome Browser snapshot of SEP3–AG (blue), SEP3^Δtet^-AG (red) and SEP3 (orange) peaks from seq-DAP-seq showing complexes bind unique sites and common sites with varying intensity. Read count are shown on the *y*-axis. Gene names (*KNUCKLES* (*KNU)* and *AGAMOUS-LIKE-18* (*AGL18)*) are given on the *x*-axis. (**C**) Comparison of SEP3–AG and SEP3 datasets. Each peak is shown as a color-coded dot, with SEP3–AG specific peaks in blue, common peaks in gray and SEP3 specific peaks in orange. Peaks were assigned as specific to a TF complex based on whether a given peak exhibited at least a 2-fold difference in normalized read coverage between datasets. (**D**) Comparison of SEP3–AG and SEP3^Δtet^-AG datasets with SEP3–AG peaks in blue, common peaks in gray and SEP3^Δtet^-AG specific peaks in pink. Peak assignment as per (C). (**E**) CFR (ratio of normalized peak coverage of SEP3–AG/SEP3), color-coded per (C). (**F**) CFR of SEP3–AG/SEP3^Δtet^-AG, color-coded per (D).

Common and specific peaks (Figure 1C and D) between two experiments (SEP3, SEP3–AG or SEP3^Δtet^-AG) were determined according to the following procedure: peaks were considered common if at least 80% of two peaks overlapped with <50% of either peak non-overlapping. Peaks with more than 50% non-overlapping were considered new peaks. These values were chosen empirically based on visual inspection of the peaks in the Integrated Genome Browser (27). The averaged normalized coverage from each experiment, divided by the peak size, was computed for each peak. Figure 1C and D were computed using R (https://www.R-project.org) and the ggplot library (28). The coverage fold reduction (CFR) was computed as the ratio between the mean normalized coverages in SEP3–AG and SEP3^Δtet^-AG seq-DAP-seq. For Figure 3A, the regions were ranked according to their CFR and the intensity of the read signal was plotted as heat map centered on the peak maximum. For Figure 3B, the curves show the average peak intensities expressed as normalized read numbers. For Figure 4F, the bound regions were grouped in deciles according to their CFR and the percentage of sequences with the enriched spacings were calculated (using the indicated threshold to detect binding sites with the TFFM models). For Supplementary Figure S8, TFFM were used to calculate the best score in each bound region in ChIP-seq (SEP3 and AG) or in the SEP3–AG seq-DAP-seq. Results were box plotted and a *t*-test was applied.

#### Position weight matrices (PWM) and transcription factor flexible model (TFFM)

For each experiment, position weight matrices (PWM) and TFFM were determined on the 600 best peaks (judged according to their averaged coverage). PWM were generated by the meme-suite, using meme-chip (29) with options -meme-minw 16, -meme-maxw 16, -meme-nmotifs 1 –meme-pal. TFFM were generated using the TFFM-framework package and the PWM obtained in the meme-chip output (30). Based on the best TFFM/PWM score obtained for each bound region, each model prediction power was assessed by AUROC (Area Under the Receiver Operating Characteristic Curve) on the set of peaks excluding the 600 peaks used in the training set against an unbound set of regions, chosen with similar GC content, size, and origin (promoter, intron, exon and intergenic) than the set of bound regions (31).

#### Determination of preferred tetramer configuration

The computational method to plot intersite spacing was described in Stigliani *et al.* (31). The script was modified to include the use of TFFM. The normalized enrichment was computed by inventorying all the configurations made of two binding sites with scores above the given threshold. Since the PWM and TFFM used in this study are quasi symmetrical, we chose to consider indifferently the binding sites orientation. We then calculated the frequency (f) of each particular spacing (Cn with 0 ≤ *n* ≤ *S*_max_) among all dimeric sites in the positive set of bound regions and in the background set. Here, n and *S*_max_, respectively, stand for the considered spacing and the maximal spacing.

$$\begin{equation*}\ {f_{i,C}} = \ \frac{{{C_i}}}{{\mathop \sum \nolimits_{k\ = \ 0}^{{S_{{\rm max}}}} {C_k}}}\end{equation*}$$

The normalized enrichment (*N*) shown in Figure 4 corresponds to the ratio between frequencies in the positive set and in the negative set for a given spacing.

$$\begin{equation*}\ {N_{i,C}} = \ \frac{{{f_{i,{c_{pos}}}}}}{{{f_{i,{c_{neg}}}}}}\end{equation*}$$

#### RNA-seq experiments

Total RNA were extracted as described from wild-type (WT), *sep1 sep2* and *sep1 sep2 sep3* mutants, in duplicate and from two independent lines of *sep1 sep2 sep3* expressing *SEP3* (20). Quality of the total RNA was validated by their 260/280 absorbance ratio and the integrity of the ribosomal RNA on gel. RNA libraries construction and sequencing were performed by GENEWIZ (USA) using Illumina HiSeq and 2 × 150 bp configuration. Between 25 and 35 million reads were obtained for each library. Mapping onto the Arabidopsis genome (TAIR10), read count per gene and statistical analysis were done using STAR (no multimapping, mismatch number < 10), FeatureCount (default parameters) and EdgeR (default parameters), respectively, available in the Galaxy platform (32). Genes were considered differentially expressed (DE) between two genotypes when the log FC was >1 or <−1 and the FDR value < 0.05. DE genes were determined for *sep1 sep2 sep3* versus *sep1 sep2*, and *sep1 sep2 sep3* expressing *SEP3* versus *sep1 sep2 sep3*. The two DE gene lists were combined to give a full list of genes differentially regulated by SEP3 in at least one comparison.

#### ChIP-seq data analysis

We used the respective datasets published by Pajoro *et al.* (33) and O’Maoiléidigh *et al.* (13) in which SEP3 and AG ChIP-seq were performed in inflorescence tissues 4 days after *AP1* induction in *pAP1:AP1-GR ap1–1 cal-1* plants for SEP3 ChIP-seq and in *pAG:AG-GFP pAP1:AP1-GR ap1–1 cal-1 ag-1* plants for AG ChIP-seq. The AG ChIP-seq peaks were directly downloaded as published and the SEP3 peaks were downloaded from the Remap database (34). SEP3 and AG common peaks were then determined by using the bedtools intersect command (35) with -f 0.8, -F 0.8 and -e options.

#### Determination of AG-regulated genes

The AG-regulated genes were determined by concatenating three lists from three different studies: two studies comparing gene expression with and without AG-GR activation in *35S::AG-GR ag-1 Ler* plants (36,37), and one study comparing gene expression in *pAG:AG-GFP pAP1:AP1-GR ap1–1 cal-1 ag-1* *Ler* plants compared to *35S:AP1-GR ap1–1 cal-1* *Ler* plants (background control) (13)

#### Determination of bound and regulated genes

For each SEP3 or AG-regulated gene, the genic region was defined using 3 kb upstream of the transcription start site and 1 kb downstream of the transcription termination site. A gene was considered bound if a ChIP-seq or a DAP-seq peak overlapped (without restriction). When dealing with genes regulated by SEP3 and AG, the directionality of the regulation was considered and only genes positively or negatively by both regulators were kept (Figure 5F and Supplementary File S1).

## RESULTS

### Determination of genome-wide binding sites of SEP3 and SEP3–AG complexes

Recent DAP-seq protocols have been used to determine the DNA binding specificity of monomeric or homo-oligomeric TFs in Arabidopsis, but with a low success rate for MADS TFs (21,22). In order to exploit the versatility of DAP-seq and apply this technique to the MADS TF family, we developed a modified DAP-seq protocol incorporating protein tagging and sequential protein complex purification called seq-DAP-seq (Figure 1A). Different epitope tags were fused to the C-terminus of the MADS TFs to reduce potential interference with protein–DNA interactions mediated by the N-terminal MADS DBD and to allow for sequential purification of the *in vitro* transcription-translation produced proteins. SEP3-FLAG, SEP3-FLAG/AG-Myc and SEP3^Δtet^-FLAG/AG-Myc complexes were purified by immunoprecipitation using either anti-FLAG or anti-FLAG and anti-Myc magnetic beads (Supplementary Figure S1). This allowed the selection of a single species of MADS complex for DNA affinity purification. A first immunoprecipitation step using anti-FLAG magnetic beads isolated SEP3-FLAG containing complexes. For purification of SEP3-FLAG/AG-Myc heterocomplexes, a second purification step using anti-Myc magnetic beads was used to isolate the desired SEP3-FLAG/AG-Myc complex and eliminate any potential contamination of SEP3 homodimers/tetramers. All DAP-seq and seq-DAP-seq experiments were performed using amplified Arabidopsis Col-0 genomic DNA, with 70–80% of reads mapped to the genome and up to 20% of reads mapped to peaks (Supplementary Figure S2). SEP3, SEP3–AG and SEP3^Δtet^-AG bound ∼2300, ∼6200 and ∼2000 genomic sites, respectively (see Figure 1B and Supplementary Figure S3 for examples). In order to quantify differences between the complexes we developed a procedure to compute a normalized read coverage for each peak present in at least one experiment. This modified procedure was necessary as automatic peak calling performed poorly in differentiating peaks that were common versus specific to the datasets (Supplementary Figure S4). A peak was assigned as specific to a given TF complex if it exhibited at least a 2-fold higher coverage for one complex as compared to the other. Using this criterion, SEP3, SEP3^Δtet^ -AG and SEP3–AG common and specific peaks were identified (Figure 1C–F).

### MADS complex binding site modeling and specificity

**Figure 2.**
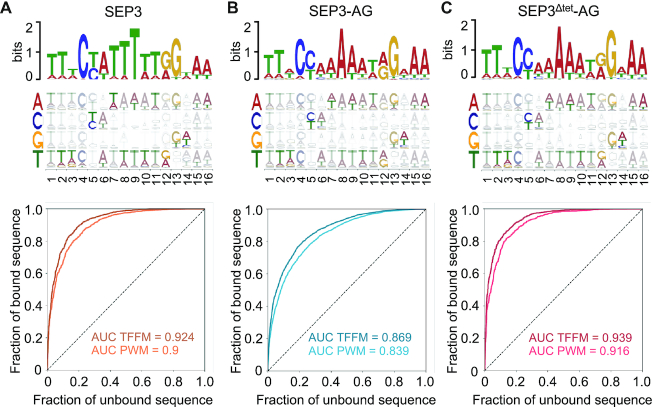
Modeling of TF binding sites. (**A**) (Top) PWM logo for SEP3 based on the top 600 bound regions. PWM computed using the MEME suite. (Middle) TFFM with dinucleotide dependencies depicted schematically. Darker colors indicate stronger dependency on the identity of the previous nucleotide. Nucleotides are color coded and shown on the *y*-axis and position given on the *x*-axis. (Bottom) PWM and TFFM were used to determine the ROC curves and AUC values. PWM are colored in light orange and TFFM in dark orange. A perfect model would account for 100% of bound regions with no false positives and yield an AUC of 1. The AUC indicate a strongly predictive model with the TFFM performing better as shown by the higher AUC scores. (**B**) (Top) PWM logo for SEP3–AG calculated as per (A). (Middle) TFFM for SEP3–AG. (Bottom) ROC curves and AUC values with PWM in light blue and TFFM in dark blue. (**C**) (Top) PWM logo for SEP3^Δtet^-AG calculated as per (A). (Middle) TFFM for SEP3–AG. (Bottom) ROC curves and AUC values with PWM in pink and TFFM in dark pink.

To decipher the specificity of each complex, we modeled their transcription factor binding sites (TFBS) using PWM and TFFM, a Hidden Markov Model-based TFBS modeling method, which accounts for dinucleotide dependencies (Figure 2) (30,38). The top 600 peaks for each complex were used for motif determination using the MEME Suite (29) and the predictive power of each model was evaluated on the total sets of bound regions based on an Area Under the Receiver Operating Curve (AUROC). AUROC analysis was performed by comparison with a set of unbound regions, with an AUROC score of 1 indicating a perfect model and 0.5 indicating a model with no predictive power. The PWM were determined for SEP3, SEP3–AG and SEP3^Δtet^-AG, revealing highly similar CArG box binding motifs for the different complexes (Figure 2) with AUROC values of 0.9, 0.839 and 0.916, for SEP3, SEP3–AG and SEP3^Δtet^-AG, respectively, indicative of highly predictive TFBS models. The AUROC values were further improved with TFFM, yielding exceptional AUROC values of 0.924, 0.869 and 0.939 for SEP3, SEP3–AG and SEP3^Δtet^-AG, respectively (Figure 2). The SEP3-derived models perform slightly less well on SEP3–AG data, suggesting that SEP3 and SEP3–AG complexes recognize somewhat different DNA binding sites (Supplementary Figure S5). In contrast, applying the TFBS models of SEP3–AG to SEP3^Δtet^-AG and vice versa demonstrated that each model performed equally as well for each dataset (Supplementary Figure S5). This is consistent with SEP3^Δtet^-AG dimer and SEP3–AG tetramer sharing the same DBD and thus recognizing the same DNA motifs. The slightly higher overall AUROC values for the SEP3^Δtet^-AG complex suggests an additional layer of specificity for SEP3–AG, not captured by the PWM or TFFM.

### Tetramerization of SEP3–AG increases DNA-binding affinity and enriches for specific intersite spacing

**Figure 3.**
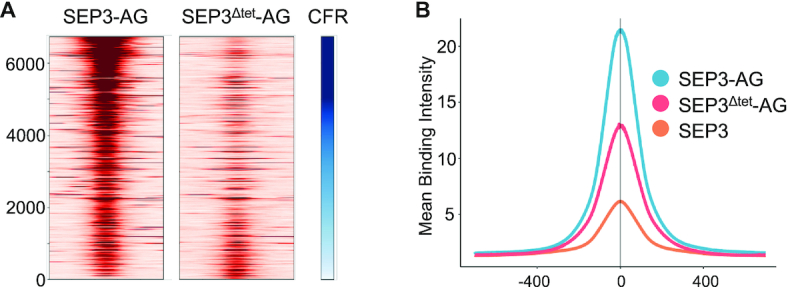
Binding comparisons for seq-DAP-seq datasets. (**A**) Heat map of read intensity for SEP3–AG (left) and SEP3^Δtet^-AG (right) with dark red indicating highest number of reads. Peaks are sorted by CFR on the *y*-axis (CFR ranging from 16.45 to 0.37, with highest CFR in dark blue); *x*-axis is a 1000 bp window for each peak. (**B**) Mean binding intensity of seq-DAP-seq peaks for SEP3–AG (blue), SEP3^Δtet^-AG (pink) and SEP3 (orange). *X*-axis is number of bp from the peak center at 0. The mean binding intensity is normalized to read count.

Comparing seq-DAP-seq datasets for tetrameric SEP3–AG and dimeric SEP3^Δtet^-AG, the tetrameric form had more bound regions and a greater proportion of mapped reads in the peaks than the dimeric form (Supplementary Figure S2). By presenting two DBDs instead of one, the tetramer would be predicted to be a higher affinity binder of DNA. This hypothesis is strongly supported by the seq-DAP-seq data (Figures 1D and F and 3). Comparing the normalized binding intensity for SEP3–AG and SEP3^Δtet^-AG demonstrates that while some sites are as strongly bound in both datasets, the dimeric SEP3^Δtet^-AG demonstrates a binding reduction at many sites (Figure 3). Examining the average binding intensity for sites common to SEP3–AG, SEP3^Δtet^-AG and SEP3 shows that the intensity of binding follows a clear trend with the tetrameric SEP3–AG complex binding most strongly to DNA, followed by the SEP3^Δtet^-AG complex and finally by the SEP3 homocomplex, which may be non-physiological as no specific function for the SEP3 homocomplex has been identified (Figure 3B).

**Figure 4.**
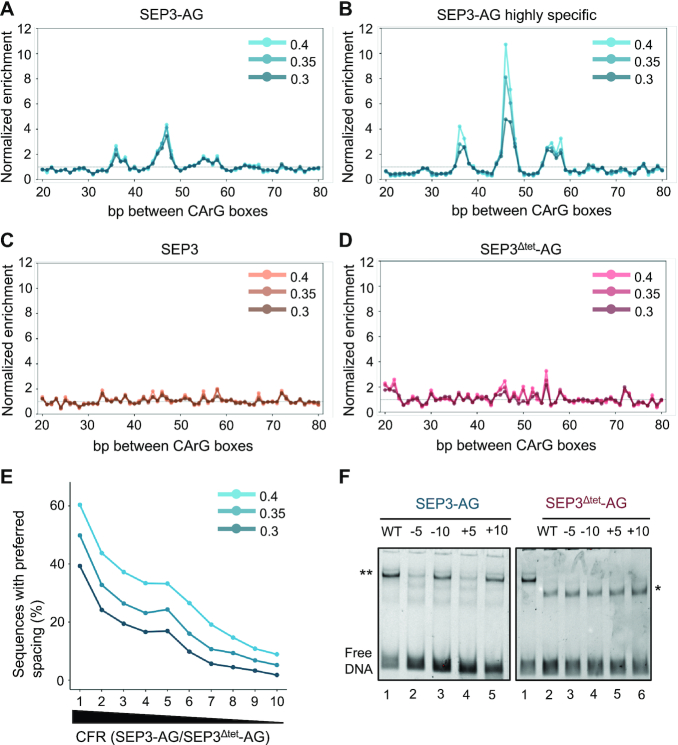
Analysis of intersite spacing for SEP3–AG, SEP3 and SEP3^Δtet^-AG. (**A**) Seq-DAP-seq data for SEP3–AG shows an over-representation of specific distances between CArG box binding sites (∼36, ∼46 and ∼56 bp). All bound regions were included in the analysis with score thresholds selected empirically as described in the ‘Materials and Methods’ section. (**B**) Peaks assigned as SEP3–AG highly specific exhibited a stronger enrichment in intersite distances. Thresholds as per (A). (**C** and **D**) SEP3 (C) or SEP3^Δtet^-AG (D) bound regions did not exhibit any intersite distance preference. (**E**) Percentage of sequences with preferred spacings in deciles of regions sorted according to the CFR SEP3–AG/SEP3^Δtet^-AG (decile 1 has strongest CFR, decile 10 the weakest). Regions for which SEP3^Δtet^-AG has strongest impact exhibited a higher frequency of preferred site spacing. (**F**) EMSA with different CArG box intersite spacing using a bound region from the *KNU* promoter. WT sequence with 45–46 bp between CArG boxes, deviations from WT spacing are labeled −5, −10, +5 and +10. SEP3–AG and SEP3^Δtet^-AG EMSAs are shown and labeled above the gel. For lane1 in SEP3^Δtet^-AG, SEP3–AG was run as a control. ** Represents the tetrameric SEP3–AG complex and * the dimeric SEP3^Δtet^-AG complex.

In order to determine whether tetramerization could play a role in addition to globally increasing binding affinity, we investigated whether the tetrameric SEP3–AG complex favors CArG boxes with specific intersite spacing, as the tetramer would be able to bind two sites concurrently. Using the optimized TFFM, we examined enrichment of intersite spacings between CArG boxes in the SEP3–AG and SEP3^Δtet^-AG bound regions. SEP3–AG bound regions revealed an enrichment of ∼36–37, 46–47 and ∼57 bp spacing between CArG box motifs (Figure 4A and B) that are not present in the SEP3^Δtet^-AG dataset nor in the SEP3 dataset, which exhibited no strong preference for any spacing (Figure 4C and D). This syntax preference became more evident when analyzing the regions highly specific for SEP3–AG (CFR > 8) (Figure 4B). Moreover, we observed a strong correlation between the intensity of the binding reduction in SEP3^Δtet^-AG and the frequency of preferred spacings in bound regions (Figure 4E). The observation that the SEP3 homocomplex did not exhibit any clear intersite spacing preference in DAP-seq, may suggest that different MADS tetrameric complexes behave differently with respect to site spacing or that the SEP3 homotetramer is less stable than the SEP3–AG heterotetramer under these conditions. EMSA studies have demonstrated intersite spacing preferences for SEP3 homotetramers, albeit on a single DNA sequence with strongly bound CArG box sites (18). Interestingly, examination of common peaks in SEP3 and AG ChIP-seq data did not reveal a preference for any intersite spacing (Supplementary Figure S6). As SEP3 and AG also form complexes with the MADS TFs, APETALA3 and PISTILLATA, the presence of these or other factors *in vivo* may mask SEP3/AG intersite distances.

To further investigate the intersite spacing preference of SEP3–AG versus SEP3^Δtet^-AG derived from seq-DAP-seq genome-wide analysis, we performed EMSAs using DNA from the promoter of *KNUCKLES* (*KNU)*. *KNU* is important for the termination of the floral meristem and acts by directly repressing the floral meristem maintenance gene, *WUSCHEL* (39). The *KNU* promoter sequence is bound in seq-DAP-seq data by SEP3–AG (Figure 1B), and has been shown to be directly regulated by AG and the SEP3–AG complex (13,20,40). Interestingly, *KNU* was not bound in AG or SEP3 ChIP-seq datasets (13,33) illustrating the variability in binding due to experimental conditions even for known direct target genes of a given TF or TF complex. The spacing of the CArG boxes in the *KNU* promoter is 45–46 bp with one site predicted to be strongly bound (CCATGTTTGG) and the second site, with two adjacent non-canonical CArG boxes (CTTCTTTGTG and TCTTCTTTGT), to be more weakly bound based on TFFM binding site prediction. We generated DNA fragments with varied spacings between the *KNU* promoter CArG box binding sites and examined the effect on SEP3–AG and SEP3^Δtet^-AG interactions by EMSA. SEP3–AG tetramer binding was very sensitive to CArG-box intersite distance, exhibiting a clear preference for 45–46 ± 10 bp spacing, with almost no tetramers bound for 45–46 ± 5 bp spacing (Figure 4F and Supplementary Figure S7A), consistent with the seq-DAP-seq analysis (Figure 4A and B). SEP3^Δtet^-AG showed no preference for any spacing, binding strongly as a dimer to all DNA fragments tested (Figure 4F). The preference for spacings that vary by ∼10 bp is likely due to the presentation of the CArG box binding sites at equivalent positions on the DNA helix which completes an integral turn every ∼10 bp. As only a single band was observed and mutation of the predicted strong binding site abrogated SEP3^Δtet^-AG, the second weaker binding site was not bound by the dimeric SEP3^Δtet^-AG complex (Supplementary Figure S7B). These results confirm that tetramerization allows access to different binding modes, in this case two-site binding with a strong spacing preference, whereas dimers preferentially bind a single high affinity site but are unable to interact with lower affinity secondary sites.

### Seq-DAP-seq identifies direct regulated targets of SEP3–AG

Seq-DAP-seq provides a global overview of potential TF binding sites genome-wide. These bound regions will include sites that are difficult to detect by ChIP-seq due to cell- or tissue-specific binding or are not bound *in vivo* due to low accessibility caused by closed chromatin structure or occupancy by other factors, for example. In addition, most bound regions *in vitro* or *in vivo* will not correspond to regulated target genes as it has been often observed that only a low proportion of binding events are linked to regulation (41–44).

**Figure 5.**
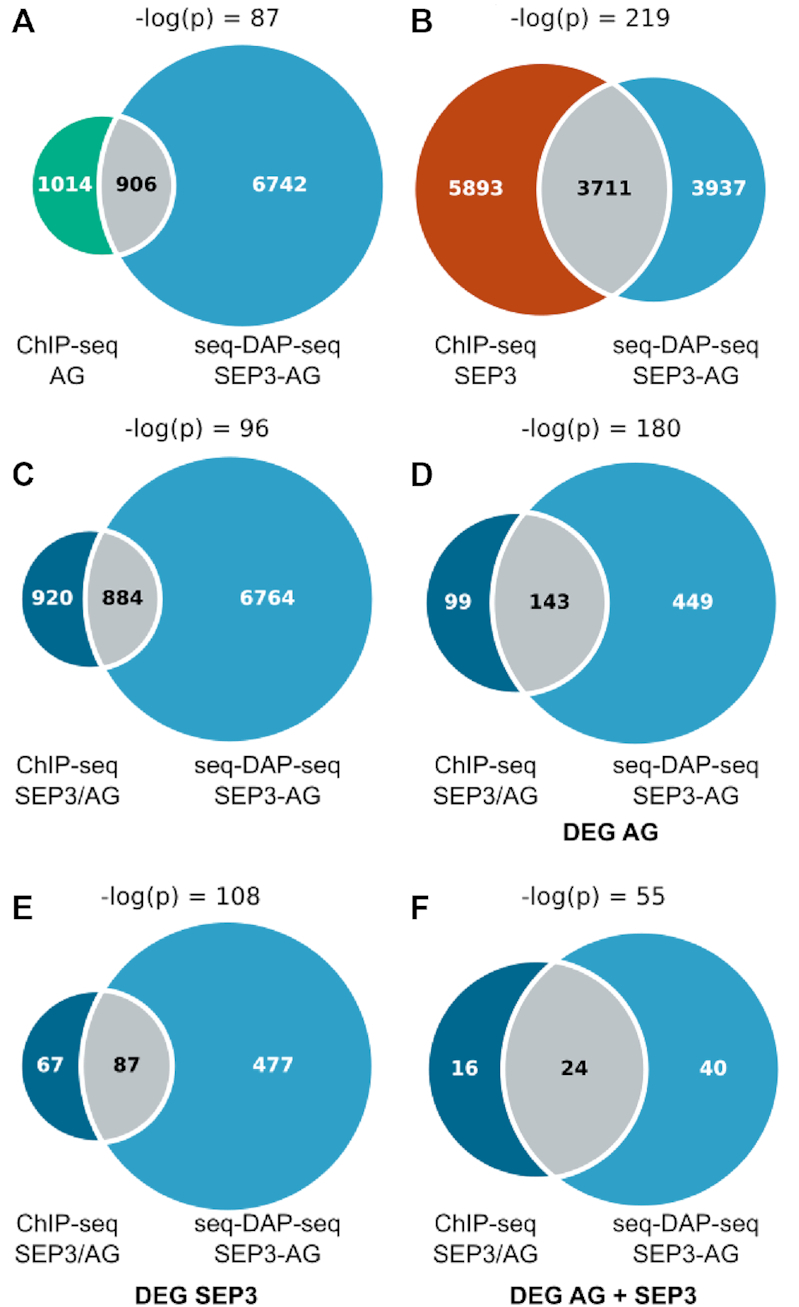
Venn diagrams for seq-DAP-seq, ChIP-seq and RNA-seq datasets. (**A**) Overlapping genes associated with peaks in AG ChIP-seq and SEP3–AG seq-DAP-seq. The AG ChIP-seq (green) and SEP3–AG seq-DAP-seq (blue) show significant overlap (gray) with –log (p) = 87. (**B**) Overlap of SEP3 ChIP-seq (brown) and SEP3–AG seq-DAP-seq (blue). Overlapping genes associated with peaks are in gray with -log (p) = 219, demonstrating highly significant overlap. (**C**) Common bound genes for AG and SEP3 ChIP-seq (dark blue) overlapped with SEP3–AG seq-DAP-seq (light blue), common genes are in graey. (**D**) Overlap of genes differentially regulated by AG (DE genes for AG were determined by combining literature experiments comparing gene expression in *35S::AG-GR ag-1* mutant with and without dexamethasone treatment (36,37) and one study comparing gene expression in *pAG:AG-GFP pAP1:AP1-GR ap1–1 cal-1 ag-1* plants compared to *35S:AP1-GR ap1–1 cal-1* plants (13)) and bound in SEP3 (33) and AG (13)ChIP-seq (dark blue) with genes differentially regulated by AG and bound in SEP3–AG seq-DAP-seq (light blue). Overlapping genes are in gray. (**E**) Overlap of genes differentially regulated by SEP3 (DE genes for SEP3 were determined by comparing *sep1 sep2 sep3* versus *sep1 sep2* and *sep1 sep2 sep3* expressing *SEP3* versus *sep1 sep2 sep3, this study*) and bound in SEP3 and AG ChIP-seq (dark blue) with genes differentially regulated by SEP3 and bound in SEP3–AG seq-DAP-seq (light blue). Overlapping genes are in gray. (**F**) Overlap of genes differentially regulated (either unregulated or down regulated in both) by AG and SEP3 and bound in SEP3 and AG ChIP-seq (dark blue) with genes differentially regulated by AG and SEP3 and bound in SEP3–AG seq-DAP-seq (light blue). Overlapping genes are in gray (gene lists are provided in Supplementary File S1).

In order to determine which of the seq-DAP-seq sites correspond to likely *in vivo* binding sites of the SEP3–AG complex and regulated target genes, we first compared the bound regions in seq-DAP-seq with those bound in ChIP-seq by AG, SEP3 or both (13,33) (Supplementary File S1). We found a significant overlap between genes associated to seq-DAP-seq regions and to ChIP-seq bound regions (Figure 5A–C). To identify regulated targets, we intersected the seq-DAP-seq and ChIP-seq binding data with the list of genes regulated by AG, SEP3 or both (Supplementary File S1) (13,14). To obtain the genes regulated by SEP3, we performed RNA-seq analyses on inflorescence meristems with young buds up to stages 10–11 of the *sep1 sep2 sep3* triple mutant compared to the *sep1 sep2* mutant or the triple *sep* mutant complemented with *SEP3* (20). Focusing on genes regulated by AG, SEP3 or both, the overlap between seq-DAP-seq- and ChIP-seq-bound genes becomes very high (60% of the ChIP-seq bound genes are present in the seq-DAP-seq list with hypergeometric test -log(p) values = 180, 108 and 55, respectively (Figure 5D–F and Supplementary File S1). This subset was chosen as it meets all the criteria for a likely direct target gene of the SEP3–AG complex- bound in both seq-DAP-seq and ChIP-seq and exhibiting differential expression in at least one RNA-seq dataset. As a control, performing the same analysis with genes bound by the SEP3 homocomplex in DAP-seq yielded a slightly less significant overlap, as predicted for the non-physiological SEP3 homocomplex (Supplementary Figure S9 and File S1). The high degree of overlap between seq-DAP-seq and ChIP-seq for SEP3–AG targets and the significant proportion of DE genes dependent on AG and/or SEP3 indicates that we are able to identify many of the binding sites by seq-DAP-seq that are bound and regulated *in vivo* by the SEP3–AG complex.

Gene ontology analysis of regions bound in both ChIP-seq and seq-DAP-seq for SEP3–AG and regulated by either AG, SEP or both shows a strong enrichment in genes important for floral meristem determinacy, flower development, organ identity, carpel development and gynoecium development (Supplementary Excel File S2). Overall, regions bound only in seq-DAP-seq have few GO terms that are enriched, with the majority of enriched GO terms corresponding to the set of common genes identified in both ChIP-seq and seq-DAP-seq experiments (Supplementary Excel File S2). Comparing seq-DAP-seq data with AG DE genes does reveal a significant enrichment in abscisic acid, apocarotenoid, sesquiterpenoid and tertiary alcohol metabolic processes, which are not enriched in ChIP-seq datasets. Whether the enrichment in these processes is physiologically relevant remains to be determined in further studies of SEP3–AG targets.

Common genes bound and regulated by SEP3–AG based on these data include well-established fourth whorl-specific genes, such as *HECATE1*, *SHATTERPROOF2*, *SUPERMAN* and *SPOROCYTELESS* (13,36,45,46) (Supplementary Figure S10 and File S1). ChIP-seq specific targets (∼21 genes) include *CRABS CLAW, HECATE2* and *SHATTERPROOF1*. Genes only bound in the seq-DAP-seq but not in the SEP3 and AG ChIP-seq include the well-characterized AG regulated target *KNU* (47), but also genes with a fourth whorl floral expression pattern, strongly suggesting they are genuine novel targets for the SEP3–AG complex, including *GAMMA-IRRADIATION AND MITOMYCIN C INDUCED 1 (GMI1), NAC DOMAIN CONTAINING PROTEIN 98* and *ATP-BINDING CASSETTE B11 (ABCB11)* (Supplementary Figure S10 and File S1). Thus, the combination of seq-DAP-seq and RNA-seq data provides a complement to ChIP-seq data and allows the identification of both known and potentially novel direct targets that may not be present in ChIP-seq experiments.

## DISCUSSION

Selection and discrimination of genomic binding sites by MADS TF complexes is fundamental to understanding how the family is able to regulate diverse processes including floral organ identity programs. The protein–protein interactions of the MADS TF family are characterized by promiscuous heterodimerization and tetramerization patterns. This is particularly true for the SEP clade, which exhibit extensive interactions with other MADS TFs and act as drivers of oligomerization (48,49). The formation of many different heteromeric MADS complexes complicates the analysis of *in vivo* binding data, particularly with respect to the differences in specificity of complexes containing some of the same partners. For example, determining whorl-specific MADS complex activity requires multiple ChIP-seq and RNA-seq datasets to identify both specific binding sites and putative direct regulatory targets for a unique MADS complex. Seq-DAP-seq has the benefits of a single purified TF complex and direct mapping to genomic DNA, thus providing a powerful tool to determine potential genome-wide binding sites of specific TF complexes as shown here for the SEP3–AG heterotetramer.

MADS TFs bind DNA as obligate dimers, however, the MIKC family of plant MADS TFs are able to tetramerize. How tetramerization may contribute to DNA binding has been difficult to quantify genome-wide, although at specific loci it has been shown to be critical for gene regulation (20). Here we demonstrate that tetramerization is able to both contribute a strong cooperativity effect for two-site binding and to globally increase the affinity of the TF complex for DNA. While these effects are difficult to detect in ChIP-seq datasets due to the complexity of binding of different MADS complexes, complementary methods including EMSA studies and, more recently, SELEX-seq, have shown a preference for specific interdistances (12,16,18). These EMSA data demonstrate that tetramerization favors specific intersite spacing with an integral number of helical turns for SEPALLATA proteins (4, 5 or 6 turns corresponding to ∼42, 52–53 or 63 bp between CArG-boxes) and short-range interactions with cooperativity effects diminishing over longer distances. Recent SELEX-seq experiments using random DNA sequences to generate TFBS models, when combined with ChIP-seq studies, suggested a preference for intersite spacing interval of ∼40–80 bp between CArG boxes for SEP3–AG complexes but with little precision on the actual spacing values (12). The seq-DAP-seq for SEP3–AG presented here allows highly precise mapping of genome wide binding and reveals three distinct intersite distance preferences, ∼36, ∼46 and ∼57 bp. The most favoured intersite distance, ∼46 bp, is present in the KNU promoter. Furthermore, we demonstrate that deviations from this distance were more poorly bound by the SEP3–AG tetramer based on EMSAs. The accuracy of seq-DAP-seq coupled with the direct mapping of bound sequences allows intersite spacing to be defined at almost the single base pair level for genomic binding sites, providing new rules governing TF–DNA interactions.

While MADS TF complexes recognize very similar CArG box sites, their binding profiles vary genome-wide based on both *in vivo* and *in vitro* experiments. Modeling TFBS using the strongest bound regions yields virtually identical DNA-binding motifs, highlighting the limitation in simple models to fully capture the complexity of DNA-binding required for proper target gene regulation. TFBS models that distinguish between TFs of the same family and account for the added specificity due to complex formation are becoming increasingly recognized as important to understanding TFBS syntax (25). An important question is to what extent a simplified *in vitro* system such as seq-DAP-seq can capture physiologically relevant binding sites. Comparing ChIP-seq data for AG and SEP3 with seq-DAP-seq data for the SEP3–AG complex reveals a high degree of overlap between *in vivo* and *in vitro* bound regions (∼50% of ChIP-seq bound regions are bound in seq-DAP-seq). This suggests that seq-DAP-seq is able to capture a large portion of *in vivo* binding sites. Previous studies have shown that many *in vivo* bound regions by SEP3 and AG do not contain CArG box-like sites. Indeed, the sites bound in ChIP-seq but not bound in seq-DAP-seq have overall weaker bound sites based on TFFM analysis, suggesting these sites may require or are dependent on additional factors for recognition by the SEP3–AG complex (Supplementary Figure S8).

In order to identify direct targets and reconstruct GRN, combining expression and binding data is essential. Extensive work cataloging the stage specific and domain specific expression profiles of DE genes during flower and floral organ development has been performed using WT, inducible systems and loss-of-function mutants (10,12–14,33,50,51). Examination of ChIP-seq and seq-DAP-seq bound regions and DE genes in the fourth whorl during carpel development reveals a high degree of overlap (Figure 5) with not only many well-characterized targets of AG and/or SEP3 including *HECATE1*, *SHATTERPROOF2, SUPERMAN* and *SPOROCYTELESS* present in ChIP-seq and seq-DAP-seq datasets, but also either ChIP-seq (e.g. *CRABS CLAW, HECATE2* and *SHATTERPROOF1*) or seq-DAP-seq (e.g. *KNU*) specific targets. Further study of seq-DAP-seq specific genes including *NAC DOMAIN CONTAINING PROTEIN 98, GMI1* and *ABCB11* will be required to determine whether these are *bonafide in vivo* direct targets of the SEP3–AG complex. However, the known SEP3–AG target, *KNU*, is present in our seq-DAP-seq data but not bound in SEP3 or AG ChIP-seq data, suggesting that seq-DAP-seq is able to identify additional regulated genes of the SEP3–AG complex. GO term analysis of seq-DAP-seq bound regions and AG regulated genes suggests additional metabolic processes including abscisic acid and secondary metabolite processes may be regulated by the SEP3–AG TF complex and warrant further investigation.

The seq-DAP-seq protocol described here provides a powerful tool to interrogate the binding of TF complexes to genomic DNA. By exploiting unique C-terminal epitope tags in the seq-DAP-seq experiments, we were able to investigate unique heteromeric MADS complexes and to unambiguously identify the genomic DNA binding sites specific for each complex. We used this novel methodology to address the question of how complex formation by MADS TFs impacts DNA binding specificity and affinity genome-wide for the SEP3 homocomplex, SEP3–AG heterotetramer and SEP3^Δtet^-AG heterodimer. Applying the same protocols to other floral organ MADS complexes will likely reveal unique interspacing and organ specific target genes for the different floral organ quartets. The data presented here helps explain MADS TF binding specificity and demonstrates the importance of tetramerization for DNA-binding and site selection. As many TFs do not act alone but form larger complexes required for DNA binding and gene regulation, the seq-DAP-seq method will enable the exploration of the complicated binding syntax of heteromeric TF complexes in a simplified and elegant *in vitro* system.

## DATA AVAILABILITY

The RNA-seq and seq-DAP-seq datasets are available under the GEO accession numbers GSE150605 and GSE150528, respectively, and a session link for the UCSD genome browser is available at https://genome.ucsc.edu/s/ArnaudStigliani/MADS. Scripts can be downloaded from https://github.com/Bioinfo-LPCV-RDF/MADS_analysis.

## Supplementary Material

gkaa729_Supplemental_Files
