# Supplementary material for: Genome-wide binding of SEPALLATA3 and AGAMOUS complexes determined by sequential DNA-affinity purification sequencing
Source: Nucleic Acids Res. 2020 Sep 5;48(17):9637–48. doi: 10.1093/nar/gkaa729 (PMC7515736; doi:10.1093/nar/gkaa729)
Supplement: gkaa729_Supplemental_Files [file gkaa729_supplemental_files.zip › Supplemental_Lai_small.pdf]

Supplemental Figure 1

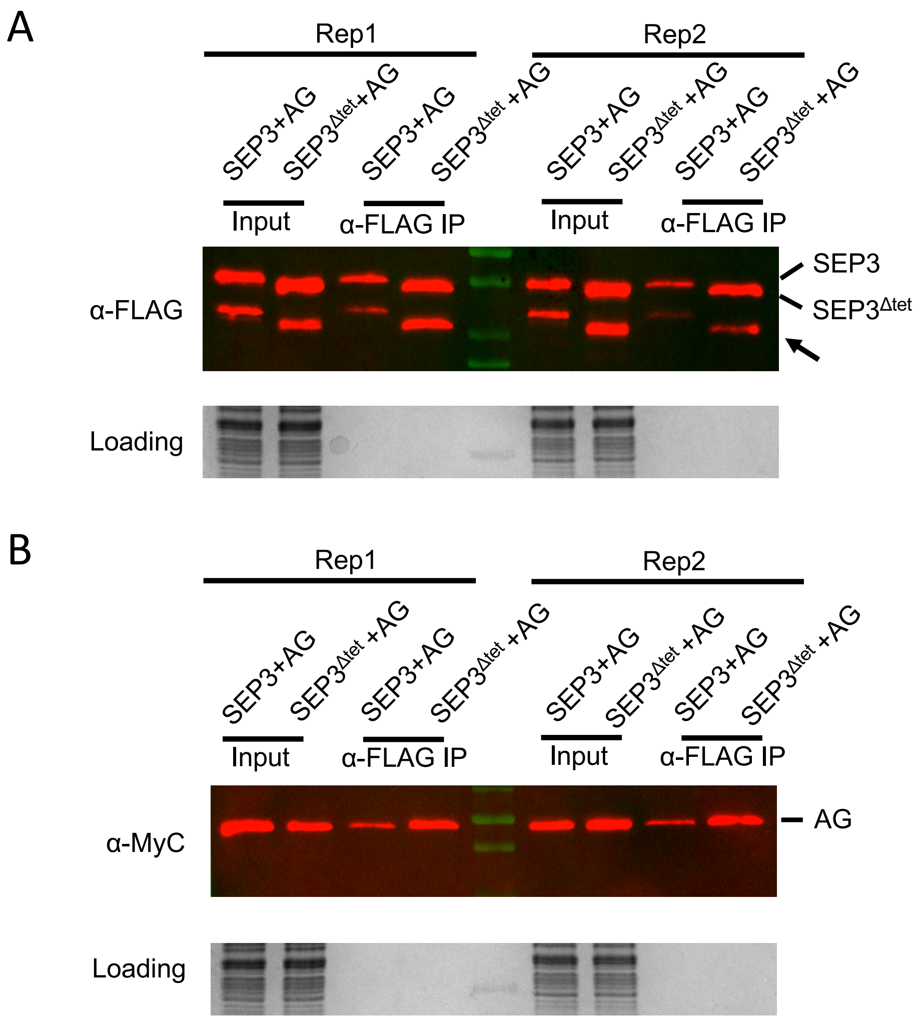

Figure S1: Western blot of SEP3-AG and SEP3$^{\Delta tet}$-AG complexes used in the seq-DAP-seq. (**A**) Anti-FLAG western blot. SEP3 and SEP3$^{\Delta tet}$ are tagged with FLAG in the C-terminus and AG with Myc in the C-terminus. The arrow indicates a degradation product of SEP3 or SEP3$^{\Delta tet}$. The protein complexes (input) are produced to a similar amount in the two reactions, more SEP3$^{\Delta tet}$-AG protein complex is retained than SEP3+AG after anti-FLAG pull-down. (**B**) Anti-Myc Western blot. For all reactions, two replicates were performed (Rep1 and Rep2). SEP3-AG and SEP3$^{\Delta tet}$-AG complexes were produced by *in vitro* transcription and translation using the same amount of plasmid DNA as input.

Supplemental Figure 2

A

	Replicates	Number of reads	% of reads mapped to genome	% of reads in peaks
Input		11267635	74.76	
SEP3	Rep1	24989437	74.48	0.53
	Rep2	17889377	74.42	1.41
	Rep3	16538597	73.92	2.24
	Rep4	20709572	73.04	10.70
SEP3$^{\Delta tet}$-AG	Rep1	15984624	74.91	6.94
	Rep2	26149053	75.69	4.80
SEP3-AG	Rep1	16314213	76.72	31.53
	Rep2	13350530	76.47	20.60
	Rep3	11806147	73.95	26.42

B

		Pearson correlation coefficient
SEP3 rep3	SEP3 rep4	0.838
SEP3 rep2	SEP3 rep4	0.744
SEP3 rep2	SEP3 rep3	0.503
SEP3 rep1	SEP3 rep4	0.815
SEP3 rep1	SEP3 rep3	0.772
SEP3 rep1	SEP3 rep2	0.684
SEP3$^{\Delta tet}$-AG rep1	SEP3$^{\Delta tet}$-AG rep2	0.909
SEP3-AG rep3	SEP3-AG rep2	0.951
SEP3-AG rep3	SEP3-AG rep1	0.951
SEP3-AG rep2	SEP3-AG rep1	0.981
SEP3-AG (merged)	SEP3 (merged)	0.433
SEP3-AG (merged)	SEP3$^{\Delta tet}$-AG (merged)	0.627

Figure S2: Next generation sequencing statistics (A) and Pearson correlation coefficient between replicates (B) of DAP-seq and seq-DAP-sep libraries. At least two replicates were performed for all experiments.

Supplemental Figure 3

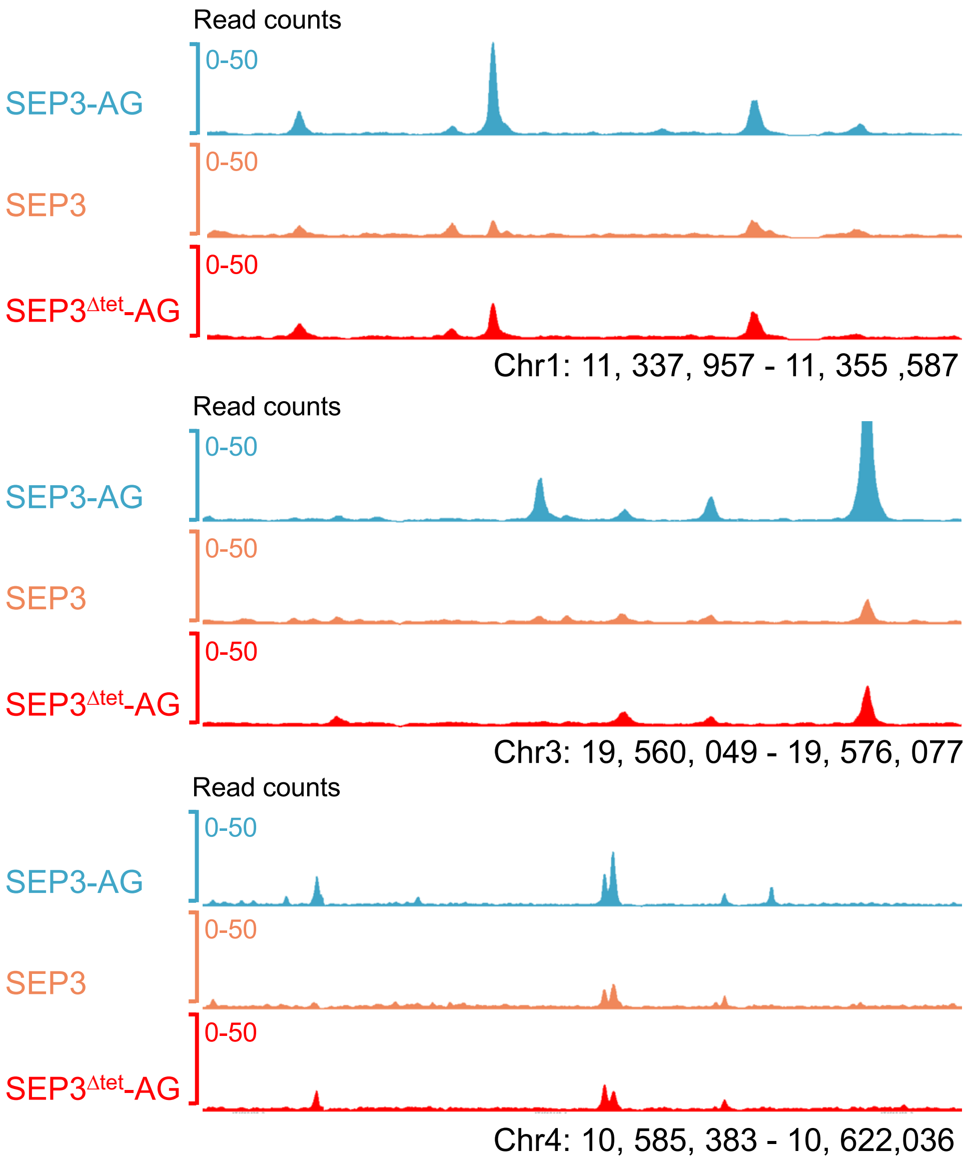

Figure S3. Representative IGB profiles of DAP-seq of SEP3, and seq-DAP-seq of SEP3-AG and SEP3$^{\Delta tet}$-AG for different chromosomal regions. Binding profiles for the complexes varies for some regions with unique sites bound or intensity of peaks changing for the different complexes.

Supplemental Figure 4

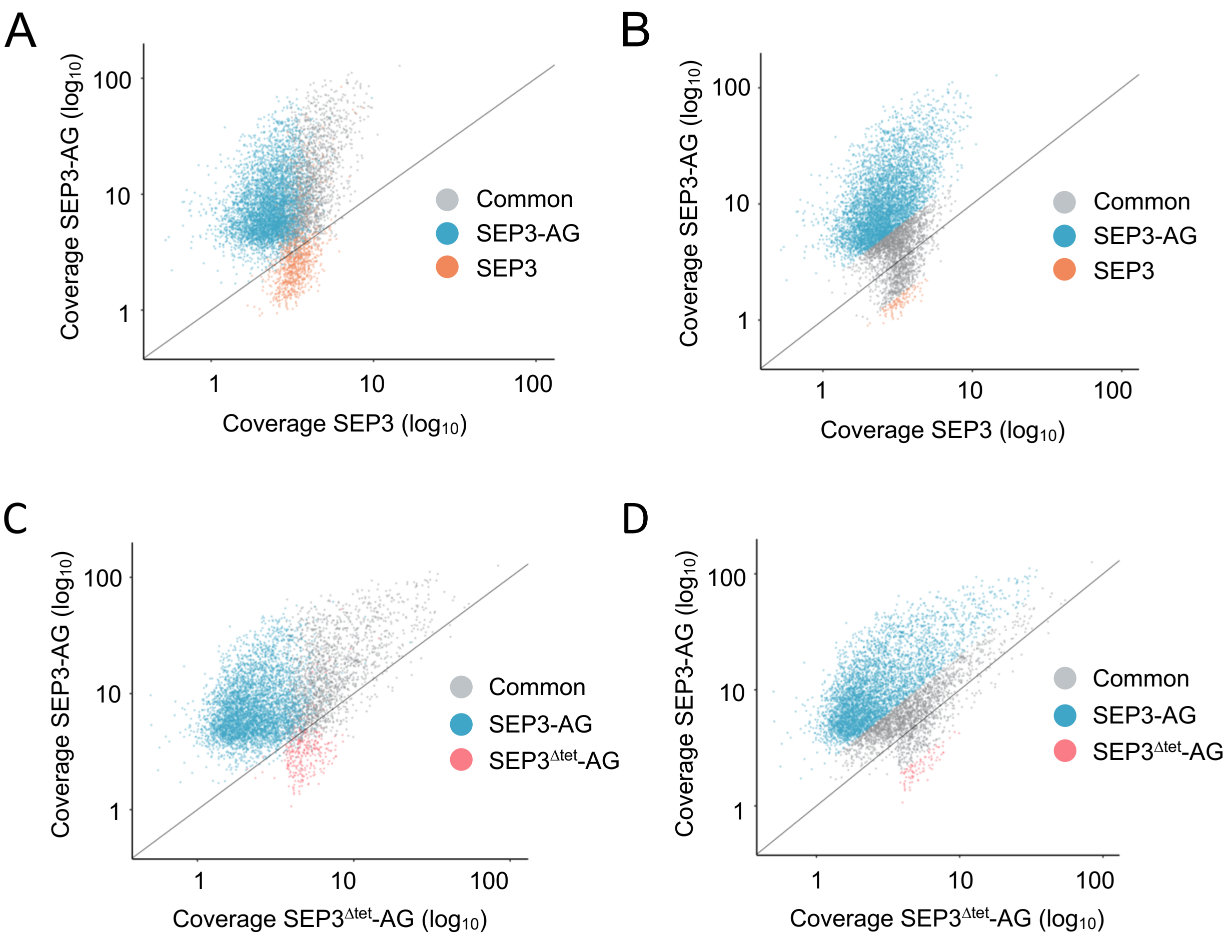

Figure S4. Comparison of peak calling using MACS2 peak caller and based on 2-fold coverage enrichment. (A) Automatic peak calling and color coding using MACS2 with SEP3-AG specific peaks in blue, SEP3 specific peaks in orange and common peaks in gray. (**B)** Peak assignment using a two-fold coverage change cut-off, with peaks colored as per (A). The automatic peak calling resulted in a number of mis-assigned peaks, which were either present in both datasets with comparable read coverage and assigned to only one or exhibited a strong fold reduction in one dataset versus the other but were assigned as common because of their high coverage. (**C** and **D**) Comparison of SEP3-AG and SEP3$^{\Delta tet}$-AG as per (A and B) with SEP3$^{\Delta tet}$-AG peaks in red.

Supplemental Figure 5

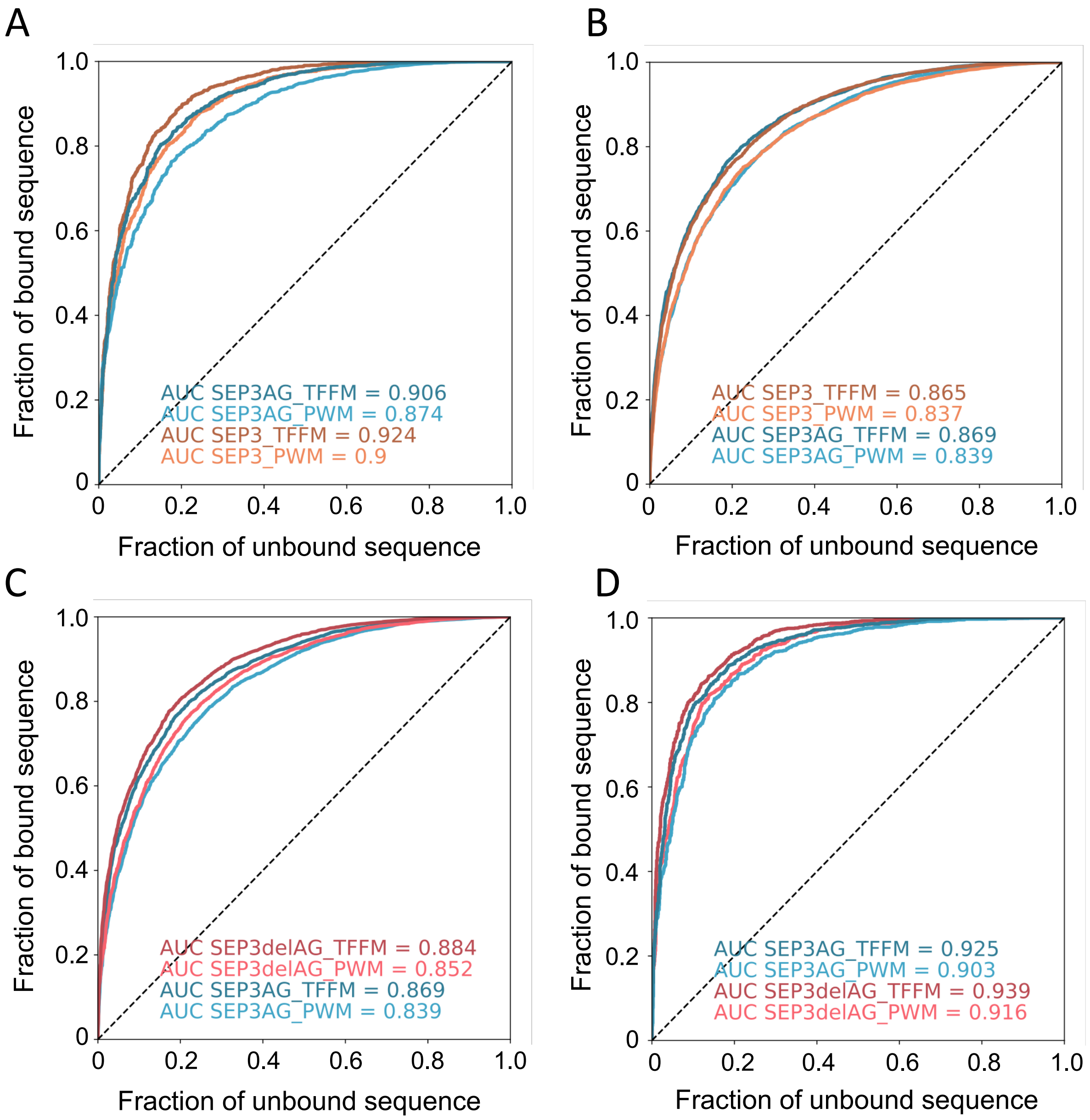

Figure S5. Comparisons of the predictive power of SEP3, SEP3-AG and SEP3$^{\Delta tet}$-AG PWM and TTFM applied to all seq-DAP-seq datasets. (A) PWM and TFFM from the top 600 SEP3 DAP-seq peaks applied to SEP3 (light and dark orange, respectively) and SEP3-AG (light and dark blue, respectively). The PWM and TFFM of SEP3 perform less well on the SEP3-AG datasets than on the SEP3 datasets. (**B)** PWM and TFFM from the top 600 SEP3$^{\Delta tet}$-AG seq-DAP-seq peaks applied to SEP3 (light and dark orange, respectively) and SEP3-AG (light and dark blue, respectively). (**C)** PWM and TFFM from the top 600 SEP3-AG DAP-seq peaks applied to SEP3$^{\Delta tet}$-AG (light and dark red, respectively) and SEP3-AG (light and dark blue, respectively). The PWM and TFFM perform similarly on the SEP3-AG and SEP3$^{\Delta tet}$-AG datasets. (**D)** PWM and TFFM from the top 600 SEP3$^{\Delta tet}$-AG DAP-seq peaks applied to SEP3$^{\Delta tet}$-AG (light and dark red, respectively) and SEP3-AG (light and dark blue, respectively). The PWM and TFFM perform similarly on the SEP3-AG and SEP3$^{\Delta tet}$-AG datasets and yield an exceptionally good model. For all comparisons the 600 peaks of the training set used to generated the PWM and TFFM models were excluded from the data in calculating the AUC scores.

Supplemental Figure 6

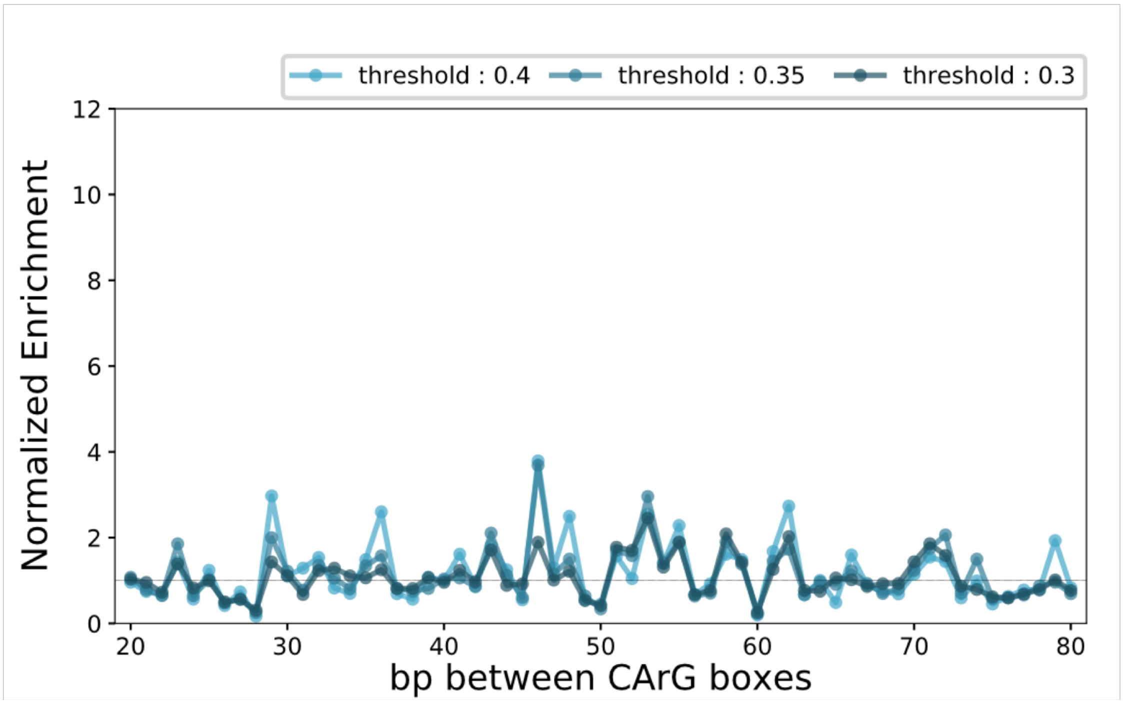

Figure S6. Analysis of intersite spacing for common peaks in SEP3 ChIP-seq and AG ChIP-seq using the TFFM derived from seq-DAP-seq of SEP3-AG. No clear enrichment intersite spacing was observed. Thresholds as per Figure 3.

Supplemental Figure 7

A

B

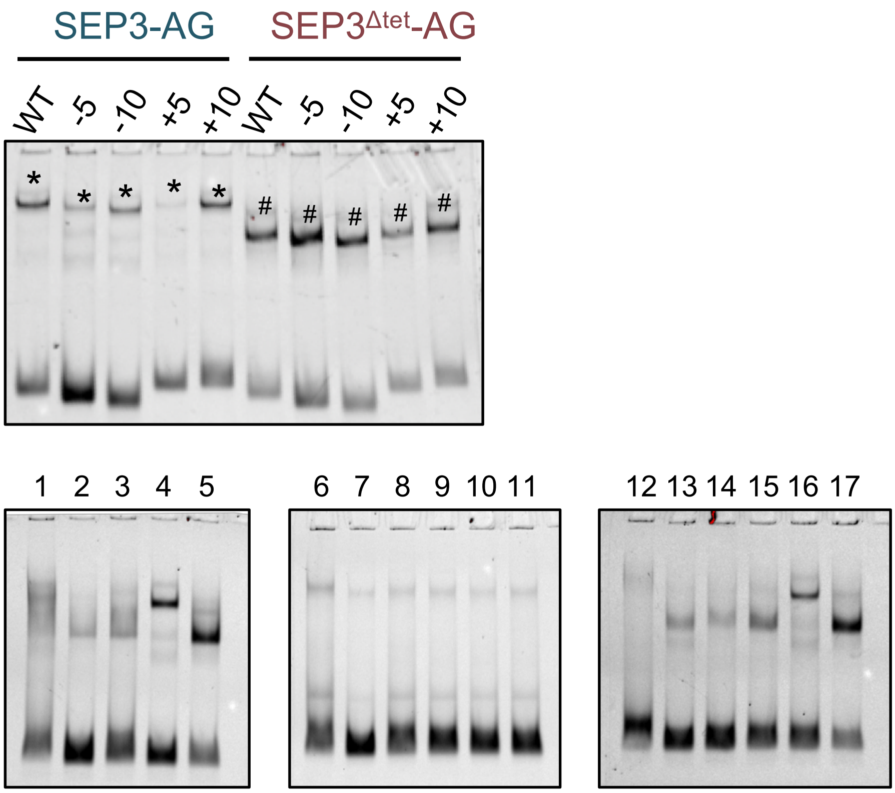

C

-5 probe:
GGTAAGAGAAACATAGAAACCTTCCATGTTTGGCAATTTCATCTTGGAACT
TACTCTCTCTTGTCTTCTTTGTGCATCACAAGAACAACAA
-10 probe:
GGTAAGAGAAACATAGAAACCTTCCATGTTTGGCAATTTCATCTTGGACTC
TCTCTTGTCTTCTTTGTGCATCACAAGAACAACAA
+5 probe:
GGTAAGAGAAACATAGAAACCTTCCATGTTTGGCAATTTCATCTTGGAAGT
CAGCTTGATTCACTCTCTCTTGTCTTCTTTGTGCATCACAAGAACAACAA
+10 probe:
GGTAAGAGAAACATAGAAACCTTCCATGTTTGGCAATTTCATCTTGGAAGT
CAGTAGCTCTTGATTCACTCTCTCTTGTCTTCTTTGTGCATCACAAGAACA
ACAA

Figure S7: EMSA of SEP3-AG and SEP3$^{\Delta tet}$-AG binding to the *KNU* promoter with 2 CArG boxes 45-46 bp apart in WT. A. EMSA of SEP3-AG and SEP3$^{\Delta tet}$-AG binding to the KNU promoter with 2 CArG boxes. Spacing was varied in 5bp increments via deletion or insertion. * indicate tetramer binding and # indicate dimer binding. SEP3-AG exhibits a clear preference for WT and +/- 10 bp spacing. **B.** Binding for *KNU* WT (left), the first CArG box site mutated (middle) and the second CArG box site mutated (right). Lanes are 1 (AG), 2 (SEP3), 3 (SEP3$^{\Delta tet}$), 4 (SEP3-AG, 5 (SEP3$^{\Delta tet}$-AG), 6 DNA alone, 7 (AG), 8 (SEP3), 9 (SEP3$^{\Delta tet}$), 10 (SEP3-AG), 11 (SEP3$^{\Delta tet}$-AG), 12 DNA alone, 13 (AG), 14 (SEP3), 15 (SEP3$^{\Delta tet}$), 16 (SEP3-AG), 17 (SEP3$^{\Delta tet}$-AG). The first CArG box is predicted to be a strong binding site based on TFFM (score 0.87) and the second site is predicted to be a weak site (0.006-0.017). **C**. Probe sequences used in **A**. CArG boxes are underlined.

Supplemental Figure 8

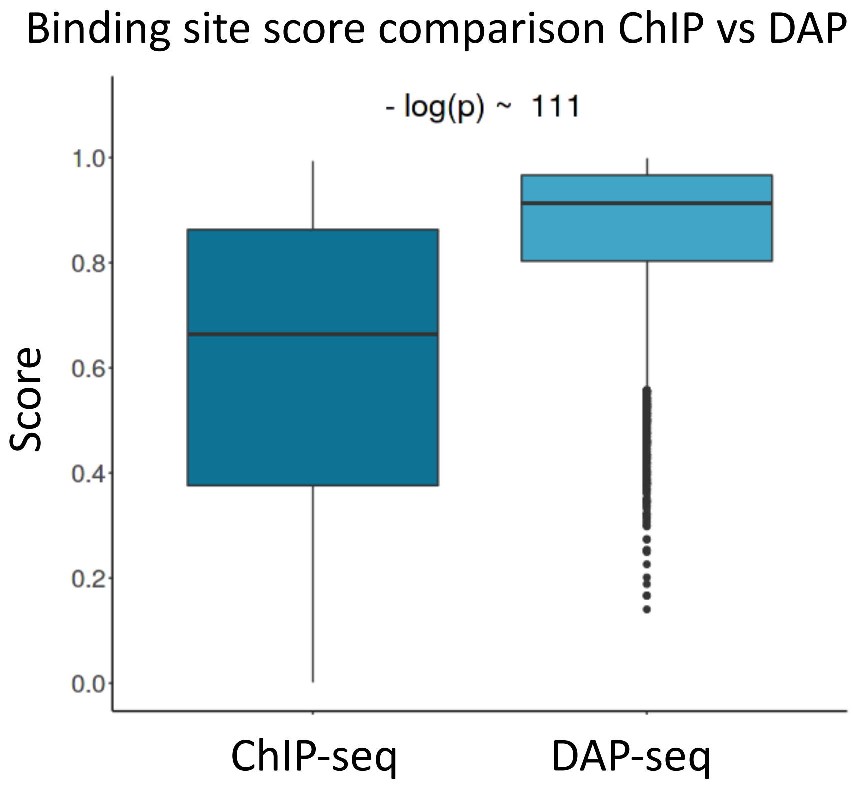

Figure S8. Comparison of binding site scores based on the SEP3-AG TFFM of peaks bound specifically in ChIP-seq by SEP3 and AG and peaks bound specifically for SEP3-AG in seq-DAP-seq. Seq-DAP-seq specific peaks exhibit a higher score than ChIP-seq specific peaks suggesting that additional factors may contribute to the *in vivo* binding patterns of the SEP3-AG complex.

Supplemental Figure 9

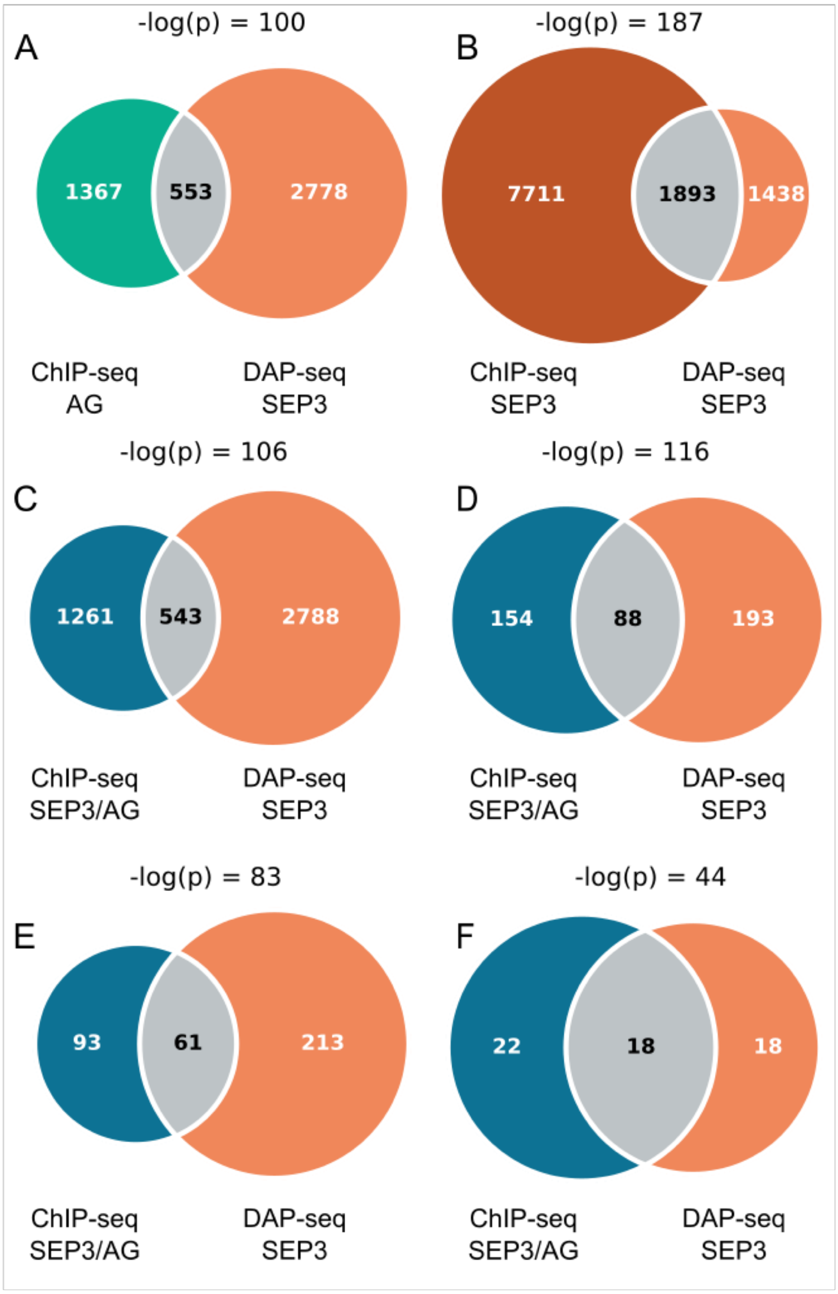

Figure S9. Venn diagrams for DAP-seq, ChIP-seq and RNA-seq datasets. (A) Overlapping genes associated with peaks in AG ChIP-seq and SEP3 DAP-seq. The AG ChIP-seq (green) and SEP3 DAP-seq (orange) show significant overlap (gray) with –log(p)=100. **(B)** Overlap between SEP3 ChIP-seq (brown) and SEP3 DAP-seq (orange). Overlapping genes are in gray with -log(p)=187. (**C)** Genes bound both in AG and SEP3 ChIP-seq (dark blue) overlapped with genes associated with SEP3 DAP-seq peaks (orange), common genes are in gray. (**D)** Overlap of genes differentially regulated by AG and bound in SEP3 and AG ChIP-seq (dark blue) with genes differentially regulated by AG and bound in SEP3 DAP-seq (orange). Overlapping genes are in gray. (**E)** Overlap of genes differentially regulated by SEP3 and bound in SEP3 and AG ChIP-seq (dark blue) with genes differentially regulated by SEP3 and bound in SEP3 DAP-seq (orange). Overlapping genes are in gray. (**F)** Overlap of genes differentially regulated by AG and SEP3 and bound in SEP3 and AG ChIP-seq (dark blue) with genes differentially regulated by AG and SEP3 and bound in SEP3 DAP-seq (orange). Overlapping genes are in gray. See Supplemental excel file S1 for list of genes.

Supplemental Figure 10

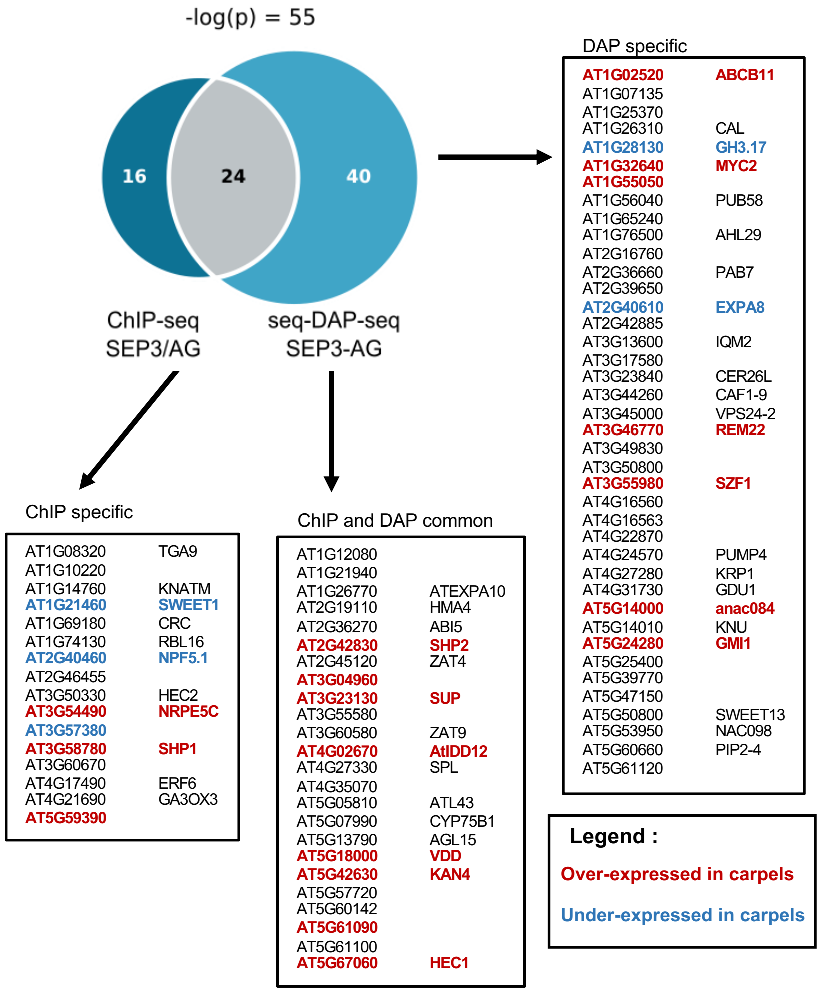

Figure S10. Venn diagram for genes regulated by AG and SEP3 (up- or down-regulated by both) and bound in ChIP-seq, seq-DAP-seq or both. SEP3-AG differentially regulated genes present specifically in ChIP-seq (AG and SEP3, dark blue), in SEP3-AG seq-DAP-seq (light blue) or in both (grey). Additional color-coding indicated genes that show a carpel expression pattern consistent with the SEP3-AG regulation (up regulated by both and with stronger expression in carpel [red] than other floral organs or down regulated and with lower expression in carpels [blue]) according to eFP browser.
